# Role of endoplasmic reticulum stress in the protective effects of PPARβ/δ activation on endothelial dysfunction induced by plasma from patients with lupus

**DOI:** 10.1186/s13075-017-1478-7

**Published:** 2017-12-06

**Authors:** Marta Toral, Rosario Jiménez, Miguel Romero, Iñaki Robles-Vera, Manuel Sánchez, Mercedes Salaices, José Mario Sabio, Juan Duarte

**Affiliations:** 1 Department of Pharmacology, School of Pharmacy, University of Granada, 18071 Granada, Spain; 2 Instituto de Investigación Biosanitaria de Granada, ibs.GRANADA, Granada, Spain; 3 CIBER of cardiovascular diseases (CIBERCV), Madrid, Spain; 4 Department of Pharmacology, School of Medicine, Autonomous University of Madrid, Research Institute Universitary Hospital La Paz (IdiPAZ), 28029 Madrid, Spain; 5 Department of Internal Medicine, Virgen de las Nieves Universitary Hospital, Granada, Spain

**Keywords:** PPARβ/δ, Systemic lupus erythematosus, Endothelial dysfunction, Endoplasmic reticulum

## Abstract

**Background:**

We tested whether GW0742, a peroxisome proliferator-activated receptor beta/delta (PPARβ/δ) agonist, improves endothelial dysfunction induced by plasma from patients with systemic lupus erythematosus (SLE) involving the inhibition of endoplasmic reticulum (ER) stress.

**Methods:**

A total of 12 non-pregnant women with lupus and 5 non-pregnant healthy women (controls) participated in the study. Cytokines and double-stranded DNA autoantibodies (anti-dsDNA) were tested in plasma samples. Endothelial cells, isolated from human umbilical cord veins (HUVECs), were used to measure nitric oxide (NO), intracellular reactive oxygen species (ROS) production, nicotinamide adenine dinucleotide phosphate (NADPH) oxidase activity, and ER stress markers.

**Results:**

Interferon-γ, interleukin-6, and interleukin-12 levels were significantly increased in plasma from patients with SLE with active nephritis (AN), as compared to both patients with SLE with inactive nephritis (IN) and the control group. The NO production stimulated by both the calcium ionophore A23187 and insulin was significantly reduced in HUVECs incubated with plasma from patients with AN-SLE as compared with the control group. Plasma from patients with IN-SLE did not modify A23187-stimulated NO production. Increased ROS production and NADPH oxidase activity were found in HUVECs incubated with plasma from patients with AN-SLE, which were suppressed by the ER stress inhibitor 4-PBA and the NADPH oxidase inhibitors, apocynin and VAS2870. GW0742 incubation restored the impaired NO production, the increased ROS levels, and the increased ER stress markers induced by plasma from patients with AN-SLE. These protective effects were abolished by the PPARβ/δ antagonist GSK0660 and by silencing PPARβ/δ.

**Conclusions:**

PPARβ/δ activation may be an important target to control endothelial dysfunction in patients with SLE.

## Background

Systemic lupus erythematosus (SLE) is an autoimmune disease characterized by acute and chronic inflammation of various tissues such as kidneys, brain, heart and vessels, often accompanied by hypertension and endothelial dysfunction among other alterations [1–3]. Numerous studies suggest that the endothelium is prominently affected during SLE, as demonstrated by the high risk of development of atherosclerosis [4, 5]. Endothelial cell dysfunction represents the earliest indicator of development of cardiovascular disease and is also a key element of SLE [6]. SLE is considered per se responsible for direct detrimental effects on the vasculature, beyond the concomitance with traditional cardiovascular risk factors, including obesity, hypertension, dyslipidemia and diabetes mellitus [7, 8]. The endothelial dysfunction in SLE is of unknown etiology.

Endothelial dysfunction is characterized by impaired nitric oxide (NO) availability and concomitant increased reactive oxygen species (ROS) generation [9]. A key mechanism of endothelial dysfunction involves the vascular production of ROS, particularly O_2_^.-^, which reacts rapidly with and inactivates NO [10]. Inflammatory responses in the endothelium induced by circulating autoantibodies and other inflammatory mediators are known to contribute to the pathogenesis of endothelial dysfunction, and numerous studies have implicated the release of cytokines in the progression of SLE [11]. It is well-stablished that pro-inflammatory cytokines increase ROS production in endothelial cells. To our knowledge, the direct effects on human endothelial cells in plasma from patients with SLE have never been studied.

Previous studies implicated abnormal unfolded protein responses, termed endoplasmic reticulum (ER) stress, in oxidative stress and endothelial dysfunction [12, 13]. ER stress has been described in peripheral blood leucocytes from patients with SLE [14]. In addition, anti-double-stranded-DNA (anti-dsDNA), the hallmark autoantibodies in SLE, induce inflammation via ER stress in human mesangial cells [15]. However, the role of ER stress in endothelial dysfunction in SLE remains undefined.

The peroxisome proliferator-activated receptors (PPARs) PPARα, PPARβ/δ, and PPARγ are members of the nuclear hormone receptor superfamily. PPARβ/δ is the least studied isoform of PPARs, and it is ubiquitously expressed in tissues [16]. Pharmacological activation of PPARβ/δ has been shown to reduce hypertension, endothelial dysfunction, and organ damage in mice with severe lupus, which was associated with reduced plasmatic anti-dsDNA autoantibodies and anti-inflammatory and antioxidant effects in target tissues, identifying PPARβ/δ as a promising target for an alternative approach to the treatment of SLE and its associated vascular damage [17]. However, it is unclear whether its protective effect on endothelial function is the indirect result of better control of both disease activity and metabolic parameters or, alternatively, a direct effect on the endothelial cells. Previous evidence indicates that PPARβ/δ agonists inhibit cytokine-induced ROS production in endothelial cells [18], at least in part, by positively regulating the antioxidant genes and eliminating excessive production of ROS. More recently it has been described that PPARβ/δ-deficient mice exhibit increased ER stress in the heart [19], and PPARβ/δ activation prevents palmitate-induced or thapsigargin-induced ER stress in the human cardiomyocyte cell line [19], skeletal muscle cells [20], and tunicamycin-induced ER stress in mouse aortic endothelial cells [12]. We hypothesized that PPARβ/δ activation might exert protective effects on human endothelial cells exposed to plasma from patients with SLE by preventing ER stress. The aim of the present study was to analyze whether plasma from patients with SLE with active and/or inactive nephritis produces endothelial dysfunction by altering human umbilical endothelial cell (HUVEC) function, and to analyze the beneficial effects of PPARβ/δ activation and the role of ER stress.

## Methods

### Patients

Consecutive non-pregnant women with SLE, who were attending our Systemic Autoimmune Diseases Unit and were ≥ 18 years of age, were included in the study. In addition, a control group matched for sex, age, and education level was included, recruited mainly among non-medical staff of our hospital that attended their annual medical health examination and were invited to participate. A smaller proportion of controls were recruited from the investigators’ acquaintances. We excluded women with SLE who had < 1 year of follow up or those who had not been reviewed at least once during the previous year since the beginning of the study. All participants were white and those with diabetes mellitus or who were smokers were excluded. Participants were evaluated using a standardized clinical interview.

Disease activity and accrual of organ damage were measured using the Safety of Estrogens in Lupus Erythematosus National Assessment version of the Systemic Lupus Erythematosus Disease Activity Index (SLEDAI) [21] and the Systemic Lupus International Collaborating Clinics/American College of Rheumatology Damage Index (SDI) [22], respectively. Lupus nephritis is one of the most severe complications of SLE. Active nephritis (AN) was defined as positive proteinuria (≥500 mg measured by 24-h urinary protein collection) and/or presence of renal failure (increase in blood urea nitrogen of more than 7.5 mmol/L and/or serum creatinine >133 μmol/L). Patients with SLE were sub-grouped into an AN group and an inactive nephritis (IN) group characterized by a history of lupus nephritis that was currently inactive and with normal renal function and SLEDAI ≤1. In the AN group, blood samples were obtained before starting treatment of lupus nephritis.

In addition, five patients fulfilled the revised classification criteria for with antiphospholipid syndrome (APS) [23] were also selected as control group with other chronic inflammatory disease. Of them, three had anticardiolipin and lupus anticoagulant antibodies, one had only anticardiolipin antibodies and one had only lupus anticoagulant antibodies. No anti-beta2-glycoprotein I antibodies were detected in plasma from these patients with APS.

### Plasma samples

Blood samples were cooled in ice and centrifuged for 20 min at 5000 g at 4 °C, and the plasma frozen at −70 °C. Plasmatic anti-dsDNA antibodies were measured as previously described [17]. Plasmatic cytokines were measured by a multiplex assay using luminex technology (Merck Millipore, Darmstadt, Germany). Plasma from all patients with SLE was negative for antiphospholipid antibodies (aPL).

### Cell culture

Endothelial cells were isolated from HUVECs using a previously reported method with several modifications [24]. The cells were cultured (Medium 199 + 20% fetal bovine serum (FBS) + penicillin/streptomycin 2mmol/L + amphotericin B 2 mmol/L + glutamine 2 mmol/L + HEPES 10 mmol/L + endothelial cell growth supplement 30 μg/mL + heparin 100 mg/mL) at 5% CO_2_ and 37 °C. HUVECs at passages 2–4 were used for all experiments. HUVECs were incubated in medium supplemented with 10% plasma from controls, patients with SLE with AN or patients with SLE with IN in the absence or presence of the PPARβ/δ agonist GW0742 (0.1 or 1 μmol/L), the chemical chaperone helping the correct folding of proteins, 4-phenylbutyric acid (4-PBA, 100 μmol/L), the non-specific nicotinamide adenine dinucleotide phosphate (NADPH) oxidase (EC number 1.6.3.1) inhibitor apocynin (10 μmol/L), or the specific NADPH oxidase inhibitor VAS2870 (10 μmol/L) for 24 h. In some experiments, cells were co-incubated with the PPARβ/δ antagonist GSK0660 (1 μmol/L) 1 h prior to the addition of GW0742. The viability of the cells after serum incubation was measured using the trypan blue technique. Briefly, both attached and floating cells were combined and stained with 0.2% trypan blue. Viable cells were counted and expressed as a proportion of cells treated with FBS alone. Moreover, in another set of experiments HUVECs were incubated with the ER-inducer tunicamycin (2 μg/mL) for 24 h [12].

### Transfection of PPARβ/δ small interfering RNA (siRNA)

Confluent HUVECs were transfected with control or PPARβ/δ-specific siRNA (pooled, validated siRNA from Dharmacon, Lafayette, CO, USA) using Lipofectamine RNAiMAX (Invitrogen) for 48 h, essentially as described previously [25].

### Quantification of NO released by DAF-2

Quantification of NO released by HUVECs was performed using the NO-sensitive fluorescent probe 4,5-diaminofluorescein (DAF-2) as described previously [24]. Briefly, cells were incubated as mentioned above for 24 h. After this period, cells were washed with PBS and then were pre-incubated with L-arginine 100 μmol/L in PBS for 5 min at 37 °C. Subsequently, DAF-2 (0.1 μmol/L) and after 2 min of incubation, either insulin (100 nmol/L) or the calcium ionophore calimycin (A23187, 1 μmol/L) was added for 30 min and cells were incubated in the dark at 37 °C. Then the fluorescence was measured using a spectrofluorimeter (Fluorostart, BMG Labtechnologies, Offenburg, Germany). The auto-fluorescence was subtracted from each value. In order to calculate NO-independent fluorescence signal, in some experiments, L-NAME (100 μmol/L) was added 15 min before the addition of L-arginine. The difference between fluorescence signal without and with L-NAME was considered NO production.

### Measurement of intracellular ROS concentration

Endothelial ROS production was measured using the fluorescent probe 5-(and-6-) chloromethyl-2′-7′-dichlorodihydrofluorescein diacetate (CM-H2DCFDA) (Invitrogen Life Technologies, Carlsbad, CA, USA) as described previously [24]. Confluent HUVECs in 96-well plates, incubated as previously explained, were then incubated with 5 μM CM-H2DCFDA for 30 min at 37 °C. The fluorescent intensity was measured at an excitation and emission wavelength of 490 nm and 545 nm, respectively, using a spectrofluorimeter (Fluorostart, BMG Labtechnologies, Offenburg, Germany).

### Protein expression and phosphorylation

Cells were incubated as described abovefor 24 h or submitted to the siRNA procedure for 48 h. Then western blotting was performed as described previously [24]. HUVEC homogenates were run on using sodium dodecyl sulphate (SDS)-polyacrilamide gel electrophoresis. Proteins were transferred to polyvinylidene difluoride (PVDF) membranes, incubated with primary mouse monoclonal anti-ATF6, rabbit polyclonal anti-phospho-IRE1α (Ser^724^), mouse monoclonal anti-CHOP (Novus Biological, Cambridge, UK), rabbit monoclonal anti-phospho-PERK(Thr^981^), rabbit polyclonal anti-phospho-IRE1α (Ser^724^), mouse monoclonal anti-CHOP (Santa Cruz Biotechnology, Santa Cruz, CA, USA), rabbit monoclonal anti-PERK (Cell Signaling Technology, MA, USA), rabbit polyclonal anti-IRE1α (Abcam, Cambridge, UK), and mouse monoclonal anti-β-actin (Sigma-Aldrich, Barcelona, Spain) antibodies overnight and with the correspondent secondary peroxidase-conjugated antibodies. Antibody binding was detected by an ECL system (Amersham Pharmacia Biotech, Amersham, UK) and densitometric analysis was performed using Scion Image-Release Beta 4.02 software (http://www.scioncorp.com).

### Reverse transcriptase-polymerase chain reaction (RT-PCR) analysis

For RT-PCR analysis, total RNA was extracted from HUVECs by homogenization and converted to complementary DNA (cDNA) by standard methods. Polymerase chain reaction was performed with a Techne Techgene thermocycler (Techne, Cambridge, UK). Quantitative real-time RT-PCR was used to analyze mRNA expression [26]. The sequences of the sense and antisense primers used for amplification are described in Table 1. Relative quantification of messenger RNA (mRNA) was assessed by RT-PCR. Quantification was performed using the ∆∆ cycle threshold (∆∆Ct) method. The housekeeping gene *Glyceraldehyde-3-phosphate dehydrogenase* (*GADPH*) and ribosomal protein L13a (RPL13a) were used for internal normalization.

**Table 1:** Oligonucleotides for real-time RT-PCR

mRNA targets	Descriptions	Sense	Antisense
*PPARβ/δ*	Peroxisome proliferator-activated receptor beta	CATTGAGCCCAAGTTCGAGT	GGTTGACCTGCAGATGGAAT
*BiP*	Binding immunoglobulin protein	CGGGATCCATGAAGCTCTCCCTGGTG	CCCAAGCTTGGGCAACTCATCTTTTTCTG
*IRE-1α*	Inositol-requiring 1 transmembrane kinase/endonuclease-1α	GAATTCCAATGCCGGCCCGGC	AAGCTTGGAGGGCGTCTGGAGTCAC
*PERK*	Eukaryotic translation initiation factor-2α kinase 3	ATTGCATCTGCCTGGTTAC	GACTCCTTCCTTTGCCTFT
*ATF-6*	Activating transcription factor-6	CAGGGAGAAGGAACTTGTGA	ACTGACCGAGGAGACGAGA
*CHOP*	CCAAT/enhancer binding protein homologous protein	ACCAAGGGAGAACCAGGAAACG	TCACCATTCGGTCAATCAGAGC
*NOX-2*	NOX-2 subunit of NADPH oxidase	CCTAAGATAGCGGTTGATGG	GACTTGAGAATGGATGCGAA
*NOX-4*	NOX-4 subunit of NADPH oxidase	AGTCAGCTCTCTCCTTTCAGG	CTTG-CCCCCTTTGAATAAAT
*Actb*	Beta actin	CGGTGAAGGTGACAGCAG	TGTGTGGACTTGGGAGAGG

### NADPH oxidase activity

NADPH-enhanced O_2_^.-^ release in homogenates from cultured HUVECs was quantified by lucigenin-enhanced chemiluminescence, as previously described [26]. Briefly, cells were incubated as mentioned above. After this period, cells were homogenized. Then, NADPH (100 μM) was added to the buffer containing the mouse aortic endothelial cell (HUVECs) homogenate suspension (30 μg protein in 500 μL), and lucigenin (5 μM) was injected automatically. NADPH oxidase activity was calculated by subtracting the basal values from those in the presence of NADPH and expressed as RLU/min/μg protein.

### Statistical analysis

Results are expressed as means ± SEM. Statistical analyses were performed using Graph Pad Prism 7 software. The Shapiro-Wilk test was used for the normally distributed continuous variables. Statistical comparisons were performed using Student’s *t* test or one-way analysis of variance (ANOVA) with Bonferroni’s procedure for post hoc analysis for parametric analysis or the Kruskal-Wallis test for non-parametric analysis. Values of *p* < 0.05 were considered significant.

## Results

### Characterization of patients’ plasma

**Fig. 1:**
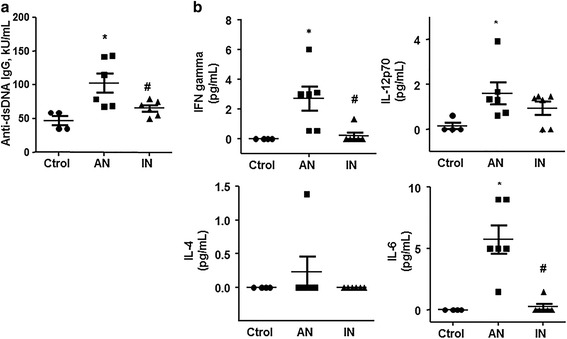
Autoantibodies and proinflammatory cytokine levels in plasma from participants. Anti-dsDNA (**a**) and interferon gamma (IFN-γ), interleukin 4 (IL-4), interleukin 6 (IL-6), and active interleukin 12 heterodimer (IL12p70) (**b**) were measured by a multiplex assay using luminex technology in plasma from patients with systemic lupus erythematosus (SLE) with active nephritis (AN) and with inactive nephritis (IN), and from healthy controls (Ctrol). Values are expressed as mean ± SEM (*n* = 5–6).^*^ *P* < 0.05 vs Ctrol. ^#^ *P* < 0.05 vs AN-SLE

We included 12 women with SLE divided into groups with AN (*n* = 6) and with IN (*n* = 6). Both groups had a similar median (IQR) age and duration of disease. All patients had SDI ≤1 and were taking hydroxychloroquine. Anti-dsDNA was increased in plasma from patients with SLE with AN as compared with controls, whereas it was significantly reduced in patients with SLE with IN (Fig. 1a). Similarly, interferon-γ, interleukin-6, and interleukin-12 levels were also significantly increased in plasma from patients with SLE as compared to the control group, and normalized in patients with SLE with IN (Fig. 1b).

### PPARβ/δ activation restores the impaired NO production induced by plasma from patients with SLE

**Fig. 2:**
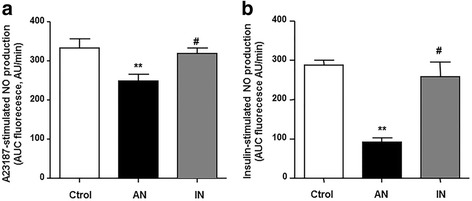
Effects of plasma incubation on stimulated nitric oxide (NO) production in human umbilical cord vein endothelial cells (HUVECs). NO production was stimulated by the calcium ionophore A23187 (**a**) or by insulin (**b**) in HUVECs incubated in plasma from patients with systemic lupus erythematosus (SLE) with active nephritis (AN) and with inactive nephritis (IN), and from healthy controls (Ctrol). NO release was estimated from the area under the curve (AUC) of the fluorescent signal of 4,5-diaminofluorescein (DAF-2) for 30 min in HUVECs stimulated with the drug. Values are expressed as mean ± SEM (*n* = 5–6). ^**^ *P* < 0.01 vs Ctrol. ^#^ *P* < 0.05 vs AN-SLE

**Fig. 3:**
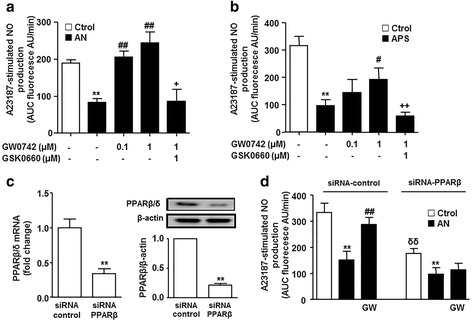
Effects of PPARβ/δ activation on endothelial nitric oxide (NO) production. NO production was stimulated by the calcium ionophore A23187 (**a**) in human umbilical cord vein endothelial cells (HUVECs) incubated in plasma from patients with systemic lupus erythematosus (SLE) with active nephritis (AN) or healthy controls (Ctrol), or in plasma from patients with antiphospholipid syndrome (APS) (**b**), in the presence and/or in absence of GW0742 and GSK0660. Values are expressed as mean ± SEM (*n* = 5–6). **c** Expression of PPARβ/δ at the level of mRNA expression by real time RT-PCR and protein by western blot in HUVECs transfected with either PPAR-β-specific siRNA (siRNA-PPAR-β) or empty vector (siRNA-control). Data are presented as gene expression normalized to *Glyceraldehyde-3-phosphate dehydrogenase* (*GAPDH*) levels or densitometric protein band and normalized to the corresponding β-actin. Results are representative of six independent experiments. ^**^ *P* < 0.05 vs siRNA-control. **d** A23187-mediated NO production in control siRNA and siRNA-PPAR-β cells incubated in plasma from patients with AN-SLE or Ctrol for 24 h, in the presence or absence of GW0742 (1 μmol/L). All data are mean ± SEM (*n* = 8). NO release was estimated from the area under the curve (AUC) of the fluorescent signal of 4,5-diaminofluorescein (DAF-2) for 30 min of stimulation. ^**^ *P* < 0.01 vs Ctrol. ^##^ *P* < 0.01*vs* without PPAR agonist. ^+^ *P* < 0.05 vs GW0742 column. ^δδ^ *P* < 0.01 vs Ctrol siRNA-control column

No significant change in cell viability was observed after incubation with plasma from patients with SLE with AN, as compared to FBS (97 ± 5% vs 100 ± 4%). Incubation of HUVECs with plasma from patients with SLE with AN reduced NO production stimulated by both the calcium ionophore A23187 (Fig. 2a), and insulin (Fig. 2b), as compared to plasma from the control group. However, plasma from patients with SLE with IN did not alter NO production stimulated by both agents. The PPAR-β/δ agonist GW0742 restored the level of NO production induced by A23187 in HUVECs exposed to plasma from patients with SLE with AN. This effect of GW0742 was abolished by co-incubation with PPARβ/δ antagonist GSK0660 (Fig. 3a). Similarly, plasma from patients with APS also reduced A23187-stimulated NO production, which was partially restored by the high concentration of GW0742 (Fig. 3b). To confirm the involvement of PPARβ/δ, in another set of experiments, HUVECs were treated with control or pooled validated PPARβ/δ siRNA. mRNA PPARβ/δ decreased by > 65% in HUVECs at 48 h post-transfection with PPARβ/δ-specific siRNA and protein decreased by > 70% (Fig. 3c), relative to control siRNA-treated cells. PPARβ/δ-specific, but not control siRNA, abolished the increase in A23187-stimulated NO production induced by GW0742 in cells incubated with plasma from patients with SLE with AN (Fig. 3d).

### PPARβ/δ activation reduced the increased ROS production induced by plasma from patients with SLE

**Fig. 4:**
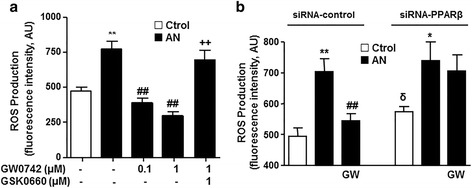
Effects of PPARβ/δ activation on intracellular Reactive oxygen species (ROS) production in human umbilical cord vein endothelial cells (HUVECs). **a** ROS production in HUVECs incubated in plasma from patients with systemic lupus erythematosus (SLE) with active nephritis (AN) or healthy controls (Ctrol), in the presence and/or in absence of GW0742 and GSK0660. Values are expressed as mean ± SEM (*n* = 5–6). **b** ROS production in control siRNA and siRNA-PPAR-β cells incubated in plasma from patients with AN-SLE or Ctrol for 24 h, in the presence or absence of GW0742 (1 μmol/L). All data are mean ± SEM (*n* = 8). ROS measured by fluorescence in CM-H2DCFDA-loaded cells. ^*^ *P* < 0.05 and ^**^ *P* < 0.01 vs Ctrol. ^##^ *P* < 0.01vs without PPAR agonist. ^++^ *P* < 0.01 vs GW0742 column. ^δ^ *P* < 0.05 vs Ctrol siRNA-control column

Intracellular ROS production measured by CM-H2DCFDA in HUVECs was increased after incubation with plasma from patients with SLE with AN (Fig. 4a). This ROS increase was inhibited by GW0742. The effect of GW0742 was abolished by blockade of PPARβ/δ by GSK0660 (Fig. 4a). When ROS production was measured in HUVECs transfected with control and PPARβ/δ siRNA and then incubated in plasma from control participants, there was greater ROS production in HUVECs with PPARβ/δ downregulation (Fig. 4b). However, in siRNA-PPARβ/δ, GW0742 did not reduce the increased ROS production stimulated by plasma from patients with SLE with AN (Fig. 4).

**Fig. 5:**
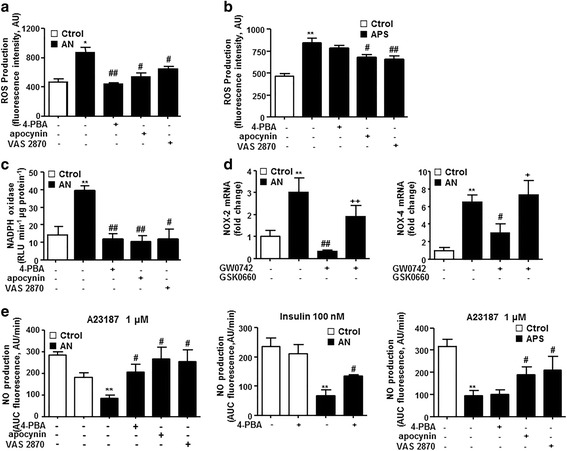
Role of endoplasmic reticulum (ER)-stress in endothelial dysfunction induced by plasma from patients with systemic lupus erythematosus (SLE) and active nephritis (AN). Reactive oxygen species (ROS) production (**a**, **b**), measured by fluorescence in CM-H2DCFDA, and nicotinamide adenine dinucleotide phosphate (NADPH) oxidase activity (**c**), measured by lucigenin-enhanced chemiluminescence, measured in human umbilical cord vein endothelial cells (HUVECs) incubated in plasma from patients with SLE with AN, or in plasma from patients with antiphospholipid syndrome (APS), or healthy controls (Ctrol), in the presence and/or in absence of 4-PBA, apocynin and VAS2870. **d** mRNA levels of NOX2 and NOX4 in HUVECs incubated in plasma from patients with SLE with AN or Ctrol, in the presence and/or in absence of GW0742 and GSK0660. **e** Nitric oxide (NO) production estimated by the area under the curve (AUC) of the fluorescent signal of 4,5-diaminofluorescein (DAF-2) after stimulation with the calcium ionophore A23187 or insulin, in HUVECs incubated in plasma from patients with SLE with AN, or APS, or Ctrol, in the presence and/or in absence of 4-PBA, apocynin and VAS2870. Values are expressed as mean ± SEM (*n* = 5–6). ^*^ *P* < 0.05 and ^**^ *P* < 0.01 vs Ctrol. ^#^ *P* < 0.05 and ^##^ *P* < 0.01vs AN group without drugs. ^+^ *P* < 0.05 and ^++^ *P* < 0.01 vs GW0742 column

Recent studies have shown that ER stress induction increases ROS production and impaired endothelial function [13]. We found that the increase in ROS production induced by SLE plasma was suppressed by a selective inhibitor of ER stress 4-PBA, and by the non-specific and the specific NADPH oxidase inhibitors apocynin and VAS2870, respectively (Fig. 5a), involving ER stress and NADPH oxidase in this increase. By contrast, the incubation with plasma from patients with APS also increased ROS production in HUVECs, but 4-PBA was unable to significantly inhibit this effect (Fig. 5b). Again, NADPH oxidase inhibition with apocynin or VAS2870 reduced the increased ROS production induced by plasma from patients with APS. NADPH oxidase is considered one of the main sources of ROS in the endothelium, which seem to be an intermediate for ER stress in endothelial dysfunction [13]. We showed that plasma from patients with SLE with AN increased NADPH oxidase activity, which was abolished by ER stress inhibition with 4-PBA, and by apocynin and VAS2870 (Fig. 5c), confirming that NADPH oxidase plays a role downstream of ER stress in these patients. Increased NADPH oxidase activity correlates with upregulation of its catalytic subunits NOX2 and NOX4 (Fig. 5d). PPARβ/δ activation reduced the increased NOX2 and NOX4 mRNA levels induced by SLE plasma (Fig. 5d)

To examine whether ER stress is involved in endothelial dysfunction induced by SLE plasma we measured NO production induced by the calcium ionophore A23187 and insulin in HUVECs incubated with 4-PBA. We found that the reduction in NO production was prevented by ER stress inhibition (Fig. 5e). By contrast, the reduced A23187-stimulated NO production induced by plasma from patients with APS was unaffected by 4-PBA, excluding ER stress on endothelial dysfunction induced by APS plasma (Fig. 5e). However, both apocynin and VAS2870 significantly improved this endothelial dysfunction, involving NAPDH oxidase as a key source of ROS in patients with APS (Fig. 5e).

**Fig. 6:**
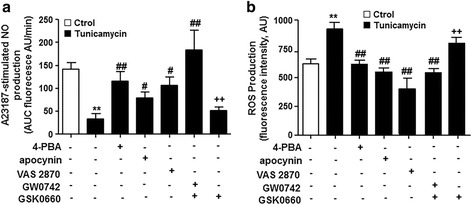
Effects of PPARβ/δ activation on endoplasmic reticulum (ER)-stress-induced endothelial dysfunction in human umbilical cord vein endothelial cells (HUVECs). Nitric oxide (NO) production stimulated by the calcium ionophore A23187 (**a**) and reactive oxygen species (ROS) production (**b**), measured by fluorescence in CM-H2DCFDA, in HUVECs incubated with ER-inducer tunicamycin for 24 h in the presence and/or in absence of 4-PBA, apocynin, VAS2870, GW0742, and GSK0660. Values are expressed as mean ± SEM (*n* = 5–6). **P < 0.01 vs healthy controls (Ctrol). #*P* < 0.05 and ##*P* < 0.01vs tunicamycin group without drugs. ++*P* < 0.01 vs GW0742 column

In our experimental conditions, tunicamycin lowered NO production triggered by A23187, which was restored by 4-PBA, apocynin, and VAS2870, involving ER stress and NADPH oxidase (Fig. 6a). GW0742 also augmented NO production in tunicamycin-treated HUVECs, whereas GSK0660 antagonized the effects of GW0742 (Fig. 6a). Similar to plasma from patients with SLE with AN, tunicamycin increased intracellular ROS production, which was abolished by ER-stress inhibition with 4-PBA, NADPH oxidase inhibition with apocynin and VAS2870, and PPARβ/δ activation (Fig. 6b). These data confirm that NADPH oxidase is an intermediate for ER stress in endothelial dysfunction.

### PPARβ/δ activation reduced the increased ER stress markers induced by plasma from patients with SLE

**Fig. 7:**
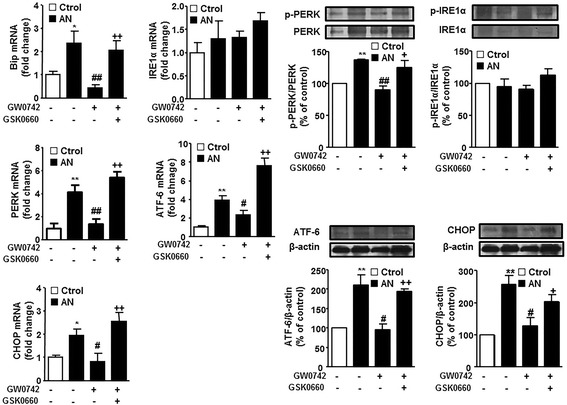
Effects of PPARβ/δ activation on endoplasmic reticulum (ER)-stress markers induced by incubation of plasma from patients with systemic lupus erythematosus (SLE) in human umbilical vein endothelial cells (HUVECs). Bip, IRE-1α, PERK, ATF-6, and CHOP mRNA levels, and protein expression of phospho-PERK, phospho-IRE-1α, ATF-6, and CHOP in HUVECs incubated in plasma from patients with SLE with active nephritis (AN) or healthy controls (Ctrol), in the presence and/or absence of GW0742 and GSK0660. Values are expressed as mean ± SEM (*n* = 5–6). ^*^ *P* < 0.05 and ^**^ *P* < 0.01 vs Ctrol. ^#^ *P* < 0.05 and ^##^ *P* < 0.01vs without PPAR agonist. ^+^ *P* < 0.05 and ^++^ *P* < 0.01 vs GW0742 column

Incubation with plasma from patients with SLE with AN increased the mRNA levels of Bip, PERK, ATF-6 and CHOP in HUVECs, as compared to the control group (Fig. 7), being without significant effect in IRE-1. Similarly, we observed increased protein expression of ATF-6 and CHOP, and increased protein phosphorylation of PERK. The PPARβ/δ agonist GW0742 reduced the increase on ER stress markers induced by SLE plasma. This effect was abolished by GSK0660 (Fig. 7).

## Discussion

Activation of PPARβ/δ has been previously reported to have beneficial effects on endothelial function in lupus mice [17]. However, in this in vivo study it is not clear if PPARβ/δ activation occurs primarily in immune cells or in the vascular endothelium, or if it is a combination of both. Herein we provide the first evidence that PPARβ/δ activation restores the impairment of NO production, induced by plasma from patients with SLE acting directly on endothelial cells via inhibition of ER stress. Moreover, this study points to PPARβ/δ and ER stress as a novel therapeutic target in human endothelial dysfunction in SLE. In addition, PPARβ/δ activation also improved endothelial dysfunction induced by plasma from patients with APS.

This study clearly demonstrates that NO production is impaired by plasma from patients with AN, stimulated by two agents, calcium ionophore A23187 and insulin, which stimulated eNOS by different pathways. The calcium ionophore A23187 activates eNOS by a calcium-dependent mechanism. However, insulin has calcium-independent vasodilator actions that are mediated by a phosphatidylinositol 3-kinase-dependent mechanism involving phosphorylation of eNOS by Akt [25, 27]. However, plasma from patients with SLE with IN did not reduce NO production stimulated by both agents. This is in contrast with data on NZBWF1 mice. In this genetic model of SLE the impaired endothelium-dependent relaxant response to acetylcholine begins before the development of proteinuria and the increase in antinuclear antibodies [28]. However, the reduced production of NO by HUVECs correlates with higher plasma cytokines (interferon gamma (IFN-γ), IL-6, IL-12) and anti-ds-DNA content in patients with SLE with AN as compared to that in patients without AN. Inflammatory responses in the endothelium induced by circulating autoantibodies and other inflammatory mediators are known to contribute to the pathogenesis of endothelial dysfunction, and numerous studies have implicated the release of cytokines in the progression of SLE [11]. It is well-established that these cytokines induce endothelial dysfunction [29, 30]. High concentrations of proinflammatory cytokines increase oxidative stress and downregulate eNOS bioactivity [31]. In fact, concentrations of IFN-γ higher than those found in SLE plasma were needed to reduce NO production in HUVECs [32], suggesting a cooperative effect among cytokines and anti-ds-DNA as responsible for endothelial dysfunction induced by plasma from patients with SLE.

aPL mediated vascular abnormalities in patients with primary APS. Patients with APS had endothelial dysfunction, as evidenced by decreased brachial artery endothelium-dependent flow-mediated dilation. Plasma samples from patients with APS revealed decreased NO availability and a pro-oxidative, proinflammatory, and pro-thrombotic state, and mice injected with aPL exibited decreased mesenteric endothelium-dependent relaxation [33]. However, aPL did not contribute to endothelial dysfunction induced by plasma from patients with SLE in our study as aPL was absent in plasma from these patients. However, incubation of HUVECs with plasma from patients with APS also reduced A23187-stimulated NO production, showing that this effect is not SLE-specific.

Interestingly, GW0742, a highly potent and selective PPAR-β/δ agonist with 200-fold higher affinity toward PPAR-β/δ than other PPAR isotypes [16], prevented the reduced NO production induced by SLE plasma, confirming a direct effect on endothelial cells. The effect of GW0742 reported herein was inhibited by GSK0660, a selective inhibitor of PPARβ/δ, confirming the specificity of this drug for this nuclear receptor. In addition, the involvement of PPARβ/δ in the protective effects of GW0742 was confirmed by silencing PPARβ/δ. In these conditions, GW0742 was unable to restore NO production stimulated by the calcium ionophore A23187. Moreover, we described for the first time that PPARβ/δ activation also increased the reduced NO production induced by plasma from patients with APS.

A key mechanism of endothelial dysfunction involves the vascular production of ROS, particularly O_2_^.-^, which reacts rapidly with and inactivates NO [10]. ROS levels are increased in the aorta [17, 34] and mesenteric arteries [35] in mice with SLE. Increased ROS levels are involved in SLE endothelial dysfunction, as in vitro incubation with the antioxidant ascorbic acid [35] or tempol [34] and in vivo treatment with tempol plus apocynin [34] normalizes endothelium-dependent relaxant responses. In our experiments, plasma from patients with SLE with AN also increased intracellular ROS production in HUVECs and GW0742 restored ROS content. This effect is derived from PPARβ/δ activation because inhibition of PPARβ/δ with GSK0660 and PPARβ/δ silencing suppressed the effect of GW0742.

The activity of the NADPH oxidase, considered the major source of O_2_^.-^ in the vascular wall, was markedly increase in mice with SLE, accompanied with an increase in mRNA level of their catalytic subunits [17, 34]. NADPH-oxidase-driven ROS production is a key event in endothelial dysfunction in SLE [17, 34]. We found that increased ROS production in HUVECs induced by SLE plasma was suppressed by incubation by both the NADPH oxidase inhibitors apocynin and VAS2870 and the ER stress inhibitor 4-PBA, involving both NADPH oxidase and ER stress as sources of intracellular ROS. Increased ROS levels were also found in HUVECs incubated with plasma from patients with APS, and seem to be involved in endothelial dysfunction evoked by this plasma, as ROS reduction with NADPH oxidase inhibitors increased A23187-stimulated NO production. However, ER stress inhibition did not alter either NO production or the ROS level in HUVECs incubated with APS plasma, suggesting different intracellular pathways involved in endothelial dysfunction evoked by plasma from patients with SLE and APS.

NADPH oxidase activity has been described as an intermediate for ER stress in vascular endothelial dysfunction [13]. In agreement with that, we also found that NADPH oxidase activity, which was increased by incubation of HUVECs with SLE plasma, was reduced by ER stress inhibition with 4-PBA. GW0742 treatment also inhibited the upregulation of NADPH oxidase subunits NOX2 and NOX4 found in HUVECs exposed to SLE plasma, suggesting ER stress inhibition. ER stress seems to be involved in the impaired A23187-stimulated and insulin-stimulated NO production induced by plasma from patients with SLE with AN, because 4-PBA improved NO levels.

To explore if GW074 inhibits ER stress in our experimental conditions we used tunicamycin to induce ER stress. This compound is a potent inhibitor of glycoprotein synthesis [36] and promotes significant ER stress in HUVECs [37]. We found that tunicamycin reduced A23187-stimulated NO production and increased ROS content. Both effects were restored by ER-stress inhibition, NADPH inhibition, and PPARβ/δ activation. These results confirm those from Cheang et al. [12] showing that the PPARβ/δ agonist GW1516 reversed tunicamycin-induced ER stress, oxidative stress, and impairment of endothelium-dependent relaxation in the mouse aorta, and NO production in mouse aortic endothelial cells.

ER stress is mediated by three ER membrane-associated proteins, PERK, ATF6, and IRE, which engage complex downstream signaling pathways, including cleavage of ATF4, activation of the eIF2α/ATF3 pathway, and splicing of X-box binding protein 1. Bone marrow mesenchymal cells from patients with SLE showed ER stress [38] evidenced by increased expression of PERK and IRE-1 [39]. However, when ER stress-related genes were analyzed in peripheral blood leucocytes from patients with SLE and compared to healthy controls, an abnormal unfolded protein response was found in patients with SLE, especially IRE-1/XBP1 and PERK/CHOP axes, with no significant change in ATF6 [14]. In our experiments, we found that SLE plasma induces ER stress in HUVECs involving only the PERK and ATF-6 pathways, without significant modification of IRE-1. PPARβ/δ activation reduced both ER stress pathways stimulated by SLE plasma. However, further investigation is needed to uncover which PPARβ/δ-responsive genes are related to protein degradation and thus regulate ER stress in the vasculature.

## Conclusions

**Fig. 8:**
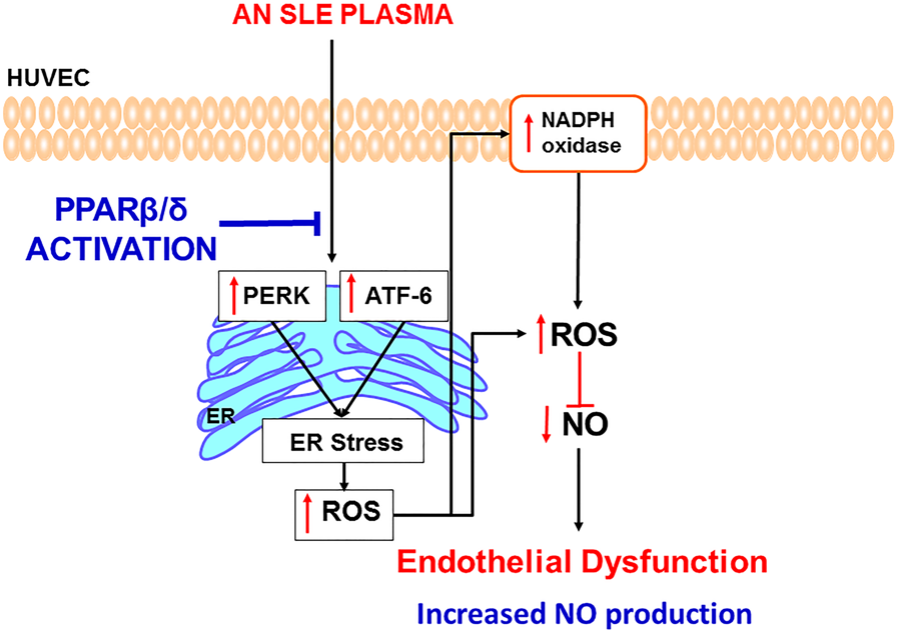
Proposed mechanism of the protective effects of PPARβ/δ activation in systemic lupus erythematosus (SLE) endothelial dysfunction. SLE plasma incubation induced endothelial dysfunction in human umbilical cord vein endothelial cells (HUVECs) by sequential activation of endoplasmic reticulum (ER)-stress and nicotinamide adenine dinucleotide phosphate (NADPH) oxidase. PPARβ/δ activation inhibited both ER stress and NADPH oxidase-driven reactive oxygen species (ROS) production reducing nitric oxide (NO) inactivation. AN, active nephritis

In conclusion, PPARβ/δ activation, by increasing NO bioavailability as a result of ER stress inhibition, may be an important target to improve endothelial dysfunction in patients with SLE (Fig. 8).

## Abbreviations

4-PBA: 4-Phenylbutyric acid
AN: Active nephritis
anti-dsDNA: Double-stranded DNA autoantibodies
aPL: Antiphospholipid antibody
APS: Antiphospholipid syndrome
ATF: Activating transcription factor
Bip: Glucose-regulated protein 78 (GRP78)/binding protein
cDNA: Complementary DNA
CHOP: CCAAT/enhancer binding homologous protein
CM-H2DCFDA: 5-(and-6-) Chloromethyl-2′-7′-dichlorodihydrofluorescein diacetate
Ct: Cycle threshold
DAF-2: 4,5-Diaminofluorescein
eIF2α: Eukaryotic initiation factor-2α
ER: Endoplasmic reticulum
GAPDH: Glyceraldehyde-3-phosphate dehydrogenase
HUVECs: Human umbilical cord vein endothelial cells
IFN-γ: Interferon gamma
IL: Interleukin
IN: Inactive nephritis
IRE1: Inositol requiring enzyme 1
NADPH: Nicotinamide adenine dinucleotide phosphate
NO: Nitric oxide
PBS: Phosphate-buffered saline
PERK: Protein kinase-like endoplasmic reticulum kinase
PPARs: Peroxisome proliferator-activated receptors
ROS: Reactive oxygen species
RT-PCR: Reverse Transcriptase-Polymerase Chain Reaction
SDI: Systemic Lupus International Collaborating Clinics/American College of Rheumatology Damage Index
siRNA: Small interfering RNA SLE, Systemic lupus erythematosus
SLEDAI: Systemic Lupus Erythematosus Disease Activity Index
